# Phylogenetic relationship and genotype variation of six Newcastle disease viruses isolated from duck in Indonesia

**DOI:** 10.14202/vetworld.2021.276-284

**Published:** 2021-01-30

**Authors:** Naimah Putri, Rahaju Ernawati, Jola Rahmahani, Suwarno Suwarno, Fedik Abdul Rantam

**Affiliations:** 1 Doctoral Program in Veterinary Science, Faculty of Veterinary Medicine, Universitas Airlangga, Surabaya, 60115, Indonesia; 2 Laboratory of Virology and Immunology, Department of Microbiology, Faculty of Veterinary Medicine, Universitas Airlangga, Surabaya, 60115, Indonesia

**Keywords:** duck, *F* gene, genotype variation, Indonesia, Newcastle disease virus, phylogenetic relationship

## Abstract

**Background and Aim:**

Newcastle disease viruses (NDVs) are frequently acquired from all ages and types of bird species. In general, ducks are considered as potential reservoirs for different genotypes of NDV and are resistant even to velogenic NDV strains. This research was conducted to genotypically and phylogenetically characterize NDV isolates collected from unvaccinated ducks from Indonesia.

**Materials and Methods:**

A total of 200 samples were collected through cloacal swabs and were inoculated in the allantoic sacs of 8-day-old specific pathogen-free eggs. Hemagglutination (HA) activity was analyzed through a HA test, and isolated viruses were characterized by reverse transcription-polymerase chain reaction targeting the complete fusion (*F*)-gene of NDV using three primer sets. One primer set was specific for the F protein cleavage site sequences of velogenic, mesogenic, and lentogenic NDV strains.

**Results:**

The results demonstrated that three isolates (NDV/Duck/B104/19, NDV/Duck/B125/19, and NDV/Duck/BK43/19) belonged to genotype VII and one (NDV/Duck/TD19/19) to genotype VI. Other isolates (NDV/Duck/A74/19 and NDV/Duck/M147/19) belonged to genotype II Class II. Based on the F protein cleavage site and the pathogenicity tests, two isolates (NDV/Duck/B104/19 and NDV/Duck/B125/19) were categorized as velogenic viruses and four (NDV/Duck/BK43/19, NDV/Duck/TD19/19, NDV/Duck/A74/19, and NDV/Duck/M147/19) as lentogenic viruses.

**Conclusion:**

The results indicate that NDVs from unvaccinated ducks from Indonesia carry various genotypes and pathotypes of NDVs; therefore, these viruses are still circulating in the environment and might pose a risk of Newcastle disease outbreak.

## Introduction

Newcastle disease (ND) is a highly contagious and fatal disease in poultry. It is caused by the ND virus (NDV) classified under the genus *Avulavirus* and species *Avian avulavirus-1* within the family Paramyxoviridae [1]. In general, waterfowl such as duck and geese are considered to be natural reservoirs for APMV-1 [2]. Based on the data from the World Organization for Animal Health (OIE), ND is notable as it causes great economic losses to the poultry industry [3]. NDV strains are categorized as highly virulent (velogenic), moderately virulent (mesogenic), or avirulent (lentogenic) based on the pathogenicity in chickens gauged by the intracerebral pathogenicity index (ICPI) and mean death time (MDT) [4]. Based on phylogenetic analysis, NDV has been genotyped into two distinct classes: Class I and class II. Class I is divided into nine genotypes and is mostly composed of avirulent viruses for chickens. Class II viruses, which occur in at least 18 genotypes, are primarily responsible for outbreaks observed in commercial poultry and include mostly virulent as well as some avirulent and vaccine strains [5-8].

NDV encompasses a diverse group of enveloped, single-stranded, and negative-sense RNA viruses with a whole-genome of approximately 15.2 kb. This genome has six open reading frames that encode for six major structural proteins: Nucleoprotein (NP), phosphoprotein (P), matrix protein (M), fusion protein (F), hemagglutinin-neuraminidase protein (HN), and the RNA-dependent RNA polymerase (L) [9,10]. Moreover, NDV has two nonstructural proteins, W and V, resulting from the differential initiation or transcriptional editing of the *P* gene mRNA [11].

The virulence of NDV strains significantly varies according to the host. Among poultry, chickens and poultry are most susceptible to NDV infections, whereas ducks and geese are least susceptible. Waterfowl such as ducks and geese are considered as natural reservoirs or carriers of NDV [12]. In recent years, NDV has caused large-scale outbreaks in Indonesia [13]. Previous studies have demonstrated that NDV genotype VII virulent strains are isolated in Bali, Indonesia [14], where it has been causing outbreaks among backyard and commercial flocks since 2009 [15,16]. NDV infections have been reported worldwide, but the infection types and virulence strength vary by region. However, the available information on the molecular characterization of NDV among ducks reared in Indonesia is scarce.

Therefore, this study was conducted to genotypically and phylogenetically characterize NDV isolates collected from unvaccinated ducks from Indonesia. The prospective outcome of our results might provide a better understanding of circulating NDVs and help in controlling the disease.

## Materials and Methods

### Ethical approval

This study was conducted according to the regulations for Research in Animal Health of Indonesian Law on Livestock and Animal Health (UU/18/2009, article 80) and samples were collected as per standard collection methods without causing any harm or stress to the animals.

### Study period and location

This study was conducted from September 2019 to February 2020 at Laboratory of Virology and Immunology, Faculty of Veterinary Medicine, Universitas Airlangga, Surabaya, Indonesia.

### Field samples

A total of 200 cloacal swab samples were collected from unvaccinated ducks from Indonesia. All samples were incubated in sterile phosphate-buffered saline (pH 7.0-7.4) containing penicillin G (2,000 units/mL), streptomycin (200 mg/mL), and mycostatin (1000 units/mL). Swab fluids were centrifuged at 3000 rpm for 20 min to inoculate 8-day-old specific pathogen-free (SPF) embryonated chicken eggs.

### Virus isolation

The allantoic cavities of 8-day-old SPF embryonated chicken eggs were inoculated with samples at 37°C for 120 h. Observations of embryonated chicken eggs were made every 12 h, and dead embryonic chicken eggs were stored in a refrigerator at 2°C. The presence of NDV was confirmed by hemagglutination (HA) and HA inhibition tests, using 1% washed chicken red blood cells, conducted as previously described [4]. Pathogenicity was evaluated by the MDT in embryonated eggs and the ICPI in 1-day-old chicks according to the standard methods described by the OIE.

### Reverse transcription-polymerase chain reaction (RT-PCR) and nucleotide sequencing

The RNA used for testing was extracted from the samples using TRIzol LS reagent (Invitrogen, Carlsbad, CA, USA) according to the manufacturer’s protocol. One-step RT-PCR (SuperScript III One-Step RT-PCR System with Platinum Taq DNA Polymerase; Life Technologies, Carlsbad, CA, USA) was employed to convert and amplify the extracted RNA samples. After RNA extraction, the *F* gene was amplified.

Primers were synthesized of specific oligonucleotides for the amplification of the *F* genes of NDV. The regions of the *F* gene of NDV isolates were amplified through PCR with primer sets F1 (forward 5′-ATCCAAGCAGGTACCCAACG-3′ and reverse 5′-AAGTCGGAGGATGTTGGCAG-3′), F2 (forward 5′-TTATTGGCGGTGTGGCTCTT-3′ and reverse 5′-TGCCGCTCAAGCAGGAATAA-3′), and F3 (forward 5′-TTTCTGCTTGAGCGGCAATA-3′ and reverse 5′-AAGCGGTAGAACGGAGGTTG-3′). These regions cover the full length of the *F* gene.

The cycling conditions were as follows: Forty cycles of 10-s denaturation at 94°C; 30-s annealing at 58°C, 53°C, or 52°C for the F1, F2, or F3 primer sets, respectively; extension for 1 min at 72 °C; and a final extension cycle at 94 °C for 10 min. After the completion of PCR, 5 μL of the reaction mixture was separated through electrophoresis on a 1% agarose gel (0.5×Tris/borate/EDTA [TBE]) containing 2 μL of ethidium bromide and subsequently visualized through UV transillumination. After purification, the PCR products were immediately sequenced.

### Phylogeny and genetic analysis

The obtained sequences were edited using the BioEdit sequence alignment editor version 7.0.9.0. Then, phylogenetic analysis was conducted utilizing the *F* gene coding sequence of NDV using the MEGA7 software [17]. Complete gene coding sequences related to the viruses investigated here and representative sequences from other genotypes were downloaded from GenBank (n=55); the accession numbers are presented in Supplementary Table-1.

**Table-1:** List of reference sequences and isolates of NDV used in this study.

Accession number	Host	Country	Year	G	MDT	ICPI	Cleavage site	Pathotyping
JQ029740	DK	China	2011	I	120	0.2	GKQGRL	Velogenic
KT381598	DK	China	2014	I	120	0.5	RKQGRL	Lentogenic
KT381599	DK	China	2014	I	120	0.2	RKQGRL	Lentogenic
KT381600 (Komarov)	DK	China	2014	II	120	0.2	GRQGRL	Lentogenic
KT381600.1	DK	China	2014	II	120	0.2	GRQGRL	Lentogenic
Y18898 (Clone 30)	CK	USA	1999	II	120	NA	GRQGRL	Lentogenic
AF309418 (B1)	CK	USA	2000	II	120	0.13	GRQGRL	Lentogenic
JF950510 (Lasota)	CK	USA	1950	II	120	0.31	GRQGRL	Lentogenic
NDV/Duck/M147/19^#^	DK	Indonesia	2019	II	110	0.12	GRQGRL	Lentogenic
NDV/Duck/A74/19^#^	DK	Indonesia	2019	II	100	0.40	GRQGRL	Lentogenic
JF950509	CK	China	2010	III	NA	NA	GRQGRL	Lentogenic
KY776604	MA	China	2006	III	NA	NA	RRQRRF	Velogenic
KY247177	CK	China	2015	III	NA	NA	RRQRRF	Velogenic
AY741404 (Herts/33)	CK	Netherlands	2004	IV	NA	NA	RRQRRF	Velogenic
MK048920	CK	Mexico	2017	V	NA	NA	RRQRRF	Velogenic
AY562987	GF	US	2002	V	NA	NA	RRQKRF	Velogenic
KF767466	CK	Spanyol	2008	V	NA	NA	RRQKRF	Velogenic
AY562988	CK	USA	1972	VI	NA	NA	RRQKRF	Velogenic
MN727300	CK	Indonesia	1951	VI	NA	NA	RRQKRF	Velogenic
KC853020	CI	China	2010	VI	NA	NA	RRQKRF	Velogenic
KT381592	PG	China	2014	VI	78	1.37	RRQKRF	Velogenic
KY042129	PG	Egypt	2015	VI	NA	NA	KRQKRF	Velogenic
NDV/Duck/TD19/19^#^	DK	Indonesia	2019	VI	90	0.37	RRQGRL	Lentogenic
JN986837	CK	Netherlands	1993	VII	NA	NA	RRQKRF	Velogenic
AY562985	CC	Indonesia	1990	VII	NA	NA	RRQKRF	Velogenic
AB605247	CK	Indonesia	2007	VII	NA	NA	RRQKRF	Velogenic
HQ697260	CK	Indonesia	2010	VII	NA	NA	RRQRRF	Velogenic
HQ697254	CK	Indonesia	2010	VII	NA	NA	RRQKRF	Velogenic
HQ697255	CK	Indonesia	2010	VII	NA	NA	RRRKRF	Velogenic
MN688614	CK	Indonesia	2014	VII	NA	NA	RRQKRF	Velogenic
MN727299	CK	Indonesia	2015	VII	NA	NA	RRQKRF	Velogenic
NDV/Duck/B104/19^#^	DK	Indonesia	2019	VII	50	1.75	RRQKRF	Velogenic
NDV/Duck/B125/19^#^	DK	Indonesia	2019	VII	45	1.60	RRQKRF	Velogenic
NDV/Duck/BK43/19^#^	DK	Indonesia	2019	VII	95	0.15	RRQGRL	Lentogenic
AF458011	CK	China	1997	VII	NA	1.94	RRQRRF	Velogenic
JN599167	PG	China	1999	VII	NA	NA	RRQKRF	Velogenic
EF175145	DK	China	2003	VII	NA	NA	RRQRRF	Velogenic
EF211811	GO	China	2006	VII	NA	NA	RRQRRF	Velogenic
DQ486859	CK	China	2007	VII	NA	NA	RRQRRF	Velogenic
GQ849007	DK	China	2008	VII	NA	NA	RRQRRF	Velogenic
KT381593	CK	China	2013	VII	70	1.73	RRQKRF	Velogenic
KT381594	DK	China	2014	VII	58	1.65	RRQKRF	Velogenic
KP189357	DK	Russia	2008	VII	NA	NA	RRQKRF	Velogenic
MF285077	CK	China	1998	VIII	NA	NA	RRQKRF	Velogenic
JX012096	CK	Malaysia	2010	VIII	NA	NA	RRQKRF	Velogenic
AF456435	GO	China	1997	IX	NA	1.84	RRQRRF	Velogenic
FJ436306	DK	China	2002	IX	NA	NA	RRQRRF	Velogenic
KT381605	DK	China	2014	IX	NA	NA	RRQRRF	Velogenic
HQ266602	CK	Madagascar	2008	XI	NA	NA	RRQRRR	Lentogenic
JX518875	CK	Madagascar	2009	XI	NA	NA	RRQRRR	Lentogenic
KC152048	GO	China	2011	XII	NA	NA	RRQKRF	Velogenic
KR732614	PC	Peru	2011	XII	NA	NA	RRQKRF	Velogenic
JN942034	OS	South Africa	1995	XIII	NA	NA	RRQKRF	Velogenic
JX393313	CX	Indonesia	1997	XIII	NA	NA	RRQKRF	Velogenic
AY865652	CK	Russia	2001	XIII	NA	NA	RRQRRF	Velogenic
KF727980	CK	India	2006	XIII	NA	NA	RRQRRF	Velogenic
MH019283	CK	Pakistan	2015	XIII	NA	NA	RRQRRF	Velogenic
JX119193	CK	Dominican R	2008	XVI	NA	NA	RRQKRF	Velogenic
KT948996	DK	Nigeria	2009	XVII	NA	NA	RRRKRF	Velogenic
KY171989	CK	Nigeria	2010	XVII	NA	NA	RRRKRF	Velogenic
KT381586.1	DK	China	2014	NA	NA	NA	GRQGRL	Lentogenic

CK=Chicken, DK=Duck, PE=Penguin, MA=Mallard, GO=Goose, OS=Ostrich, CX=Culex, CI=Crested ibis, GF=Gamefowl, PC=Peacock, NA=Not available, G=Genotype, #=Isolates in this study.

Lasota, Komarov, B1 strains are attenuated vaccine strains that widely used in Indonesia

The maximum likelihood (ML) method based on the general time-reversible model with a discrete gamma distribution (five categories [+G]) was employed for both trees. In the final analysis, there was a total of 1662 nucleotide positions. A phylogenetic tree was built using the ML method and ­employing a bootstrap resampling process (1000 replications) to evaluate the robustness of individual nodes of phylogeny. For all analyses, complete deletion was employed as the treatment for the missing data. The current criteria for the NDV classification of genotype and sub-genotype identification were followed in this study [18].

## Results

### Pathogenicity test and F gene RT-PCR

**Figure-1:**
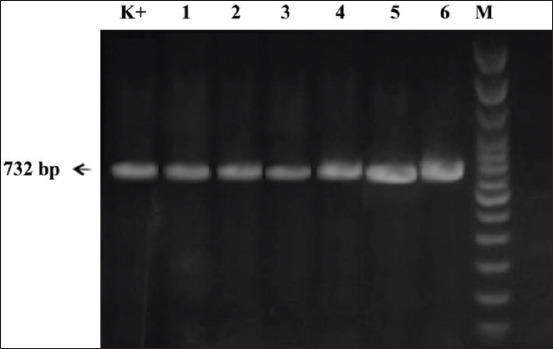
*F1* gene amplification results. Reverse transcriptase-polymerase chain reaction product size of 732 bp. The amplicons were electrophoresed in 1.5% agarose gel. Lanes: M- molecular size marker, Lane 1-6 are Newcastle disease virus (NDV) field isolates, Lane K+ NDV/Lasota (used as positive control).

**Figure-2:**
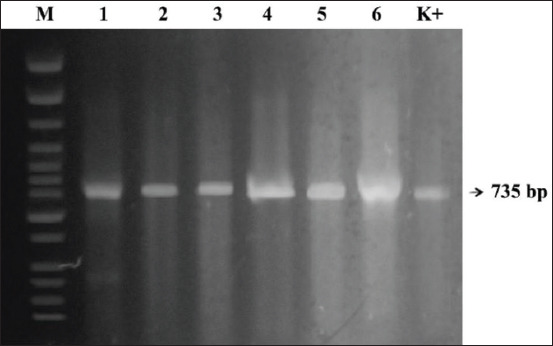
*F2* gene amplification results. Reverse transcriptase-polymerase chain reaction product size of 735 bp. The amplicons were electrophoresed in 1.5% agarose gel. Lanes: M- molecular size marker, Lane 1-6 are Newcastle disease virus (NDV) field isolates, Lane K+ NDV/Lasota (used as positive control).

**Figure-3:**
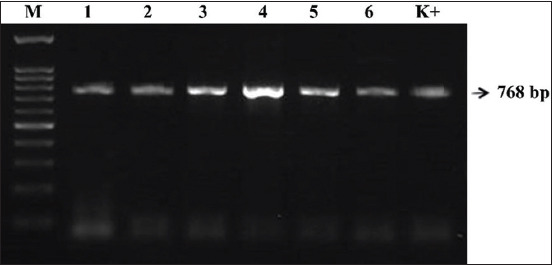
*F3* gene amplification results. Reverse transcriptase-polymerase chain reaction product size of 768 bp. The amplicons were electrophoresed in 1.5% agarose gel. Lanes: M- molecular size marker, Lane 1-6 are Newcastle disease virus (NDV) field isolates, Lane K+ NDV/Lasota (used as positive control).

In 2019, six NDV strains were isolated, namely, NDV/Duck/B104/19, NDV/Duck/B125/19, NDV/Duck/TD19/19, NDV/Duck/BK43/19, NDV/Duck/A74/19, and NDV/Duck/M147/19 (Table-1). Four isolates (NDV/Duck/TD19/19, NDV/Duck/BK43/19, NDV/Duck/A74/19, and NDV/Duck/M147/19) had an MDT of 85-110 h and ICPI of 0.12-0.43 and were thus classified as lentogenic or avirulent NDVs. Conversely, two isolates (NDV/Duck/B104/19 and NDV/Duck/B125/19) had an MDT of 35-48 h and ICPI of 1.65-1.8 and were classified as velogenic (virulent) (Table-1). Using the previously described primer pairs F1, F2, and F3, the full length of the *F* gene (1662 bp) was amplified in all isolates (Figures-1-3).

### Genetic analysis of F gene NDV

Two isolates (NDV/Duck/B104/19 and NDV/Duck/B125/19) contained the amino acid sequences RRQKRF at positions 112-117 at the C-terminus of the F1 protein, indicating that they are velogenic NDV strains. Conversely, four isolates (NDV/Duck/BK43/19, NDV/Duck/TD19/19, NDV/Duck/A74/19, and NDV/Duck/M147/19) were considered to be lentogenic with amino acid sequences GRQGRL at these positions. The nucleotide and predicted amino acid sequences of the *F* gene of NDVs were aligned; then, aligned sequences were used in pairwise comparisons, and the percentages of nucleotide sequence identities were determined (Table-2).

**Table-2:** Percentage of nucleotide sequence identities.

Isolate	1	2	3	4	5	6	7	8
Lasota	100%							
Komarov	97%	100%						
NDV/Duck/A74/19	99%	97%	100%					
NDV/Duck/M147/19	98%	96%	99%	100%				
NDV/Duck/B104/19	88%	88%	88%	88%	100%			
NDV/Duck/B125/19	88%	88%	88%	88%	100%	100%		
Duck/BK43/19	91%	89%	91%	90%	87%	87%	100%	
NDV/Duck/TD19/19	89%	88%	89%	87%	86%	86%	93%	100%

The percentage of nucleotide sequence similarity of the *F* gene of NDV between the LaSota vaccine strain and the obtained samples was in the range of 88%-99%. The NDV/Duck/A74/19 isolate exhibited the highest percentage of nucleotide sequence similarity, whereas two isolates (NDV/Duck/B104/19 and NDV/Duck/B125/19) demonstrated the lowest. In addition, the percentage of nucleotide sequence similarity between the Komarov vaccine strain and the samples was in the range of 88%-97%. The NDV/Duck/A74/19 isolate exhibited the highest percentage of nucleotide sequence, whereas three isolates (NDV/Duck/B104/19, NDV/Duck/B125/19, and NDV/Duck/TD19/19) demonstrated the lowest.

**Figure-4:**
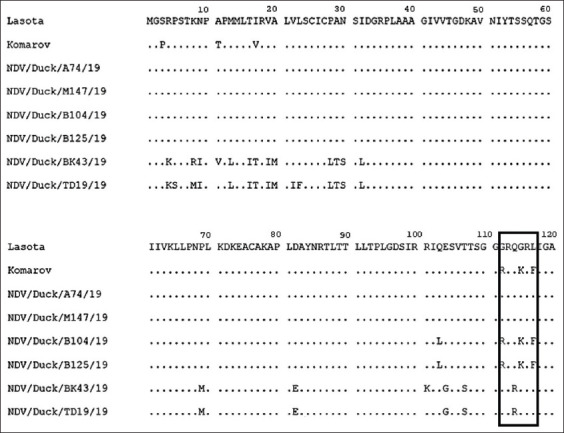
Partial deduced amino acid sequences alignment of *F* gene of NDV strains in comparison to selected strain from GenBank. Currently, part of amino acid of *F* gene from position 1 to 120 is shown. Cleavage site (black box) is indicated.

In this study, the comparative analysis of the amino acid sequences of the F protein between the isolates and vaccines used in Indonesia revealed several substitutions for amino acid residues. The mutations of the amino acid sequence of the F protein are presented in Figure-4.

### Phylogenetic analysis

**Figure-5:**
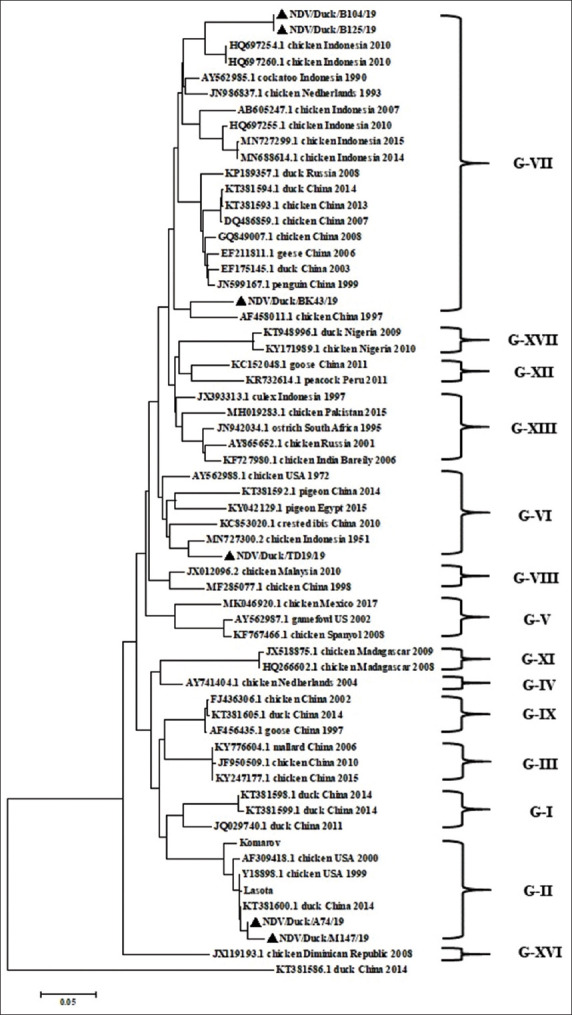
Phylogenetic relationships of the fusion gene isolates with previously published GenBank sequences for reference. The tree was constructed with bootstrap trial of 1000 replicates. The isolates in this study, respectively, were highlighted in bold and black triangle (▴). The three duck-origin NDV isolates (NDV/Duck/B104/19, NDV/Duck/B125/19, and NDV/Duck/BK43/19) belong to genotype VII. Two isolates (NDV/Duck/A74/19 and NDV/Duck/M147/19) belong to genotype II, and NDV/Duck/TD19/19 belong to genotype VI.

The phylogenetic analysis conducted using the coding sequence of the *F* gene revealed that the six NDV isolates clustered into three different Class II genotypes (Figure-5). Based on their sequences, two isolates (NDV/Duck/B104/19 and NDV/Duck/B125/19) clustered with isolates from Indonesia, which were first isolated in 2010, and belonged to genotype VII. The NDV/Duck/BK43/19 isolate clustered with a chicken virus, which was isolated in China in 1997, and belonged to genotype VII. Interestingly, the other two isolates (NDV/Duck/M147/19 and NDV/Duck/A74/19) clustered with samples from ducks from China in 2014 and formed a separate monophyletic branch within genotype II. The NDV/Duck/TD19/19 isolate clustered with isolates from chickens from Indonesia in 1951 and belonged to genotype VI. Furthermore, all isolates were not closely phylogenetically related to the vaccine strains of genotype II, such as Clone 30, LaSota, Komarov, B1, and Herts/33.

The mean evolutionary distances for the full-length F nucleotide sequences between recent and historical virulent NDV isolates were determined using MEGA 7 software; the distances between isolate NDV/Duck/A74/19 and traditional vaccine genotype II were 0.7% and 3.2%, respectively (Table-3). These results indicate the evolutionary diversity between traditional vaccine and isolates NDV/Duck/B104/19 and NDV/Duck/B125/19 in genotype VII (13.5% and 13.6%, respectively).

**Table-3:** Estimates of evolutionary distances between recent isolates and traditional vaccine.

Isolate	1	2	3	4	5	6	7	8
Lasota (G II)		0.005	0.002	0.004	0.015	0.015	0.012	0.015
Komarov (G II)	0.028		0.005	0.007	0.015	0.015	0.013	0.015
NDV/Duck/A74/19 (G II)	**0.007**	**0.032**		0.003	0.014	0.014	0.011	0.015
NDV/Duck/M147/19 (G II)	0.022	0.047	0.015		0.015	0.015	0.013	0.016
NDV/Duck/B104/19 (G VII)	0.135	0.136	0.129	0.134		0.000	0.017	0.018
NDV/Duck/B125/19 (G VII)	0.135	0.136	0.129	0.134	0.000		0.017	0.018
NDV/Duck/BK43/19 (G VII)	0.100	0.116	0.096	0.113	0.149	0.149		0.010
NDV/Duck/TD19/19 (G VI)	0.125	0.129	0.129	0.145	0.163	0.163	0.074	

The distances were inferred from the full-length nucleotide F protein gene sequences. Standard error estimate(s) are shown above the diagonal in parentheses and were obtained by bootstrap (1000 replicates). There were a total of 1662 positions in the final data set. Evolutionary analysis was conducted in MEGA7 [17]. Values indicating low genetic distance between recent isolates and traditional vaccine (0.7% and 3.2%) are in bold and those indicating greater genetic distance (13.5% and 13.6%) are underlined

## Discussion

Historically, ND has been endemic to Indonesia. However, in 2009 and 2010, ND outbreaks were reported, which caused up to 10%-80% mortality [13]. Over 241 different bird species worldwide have been reported to be susceptible to NDV [19]. Waterfowl have been known to be natural reservoirs of NDVs, which mostly belong to lentogenic strains [4]; however, investigation of unvaccinated domestic ducks for avirulent strains of NDV has never been conducted in Indonesia.

In this study, six strains isolated from Indonesia in 2019 were genotypically and pathotypically characterized. Two isolates (NDV/Duck/B104/19 and NDV/Duck/B125/19) met the OIE criteria for a velogenic NDV. This indicates that NDV from waterfowl may be highly pathogenic to terrestrial birds and waterfowl and may be transmitted between them. Four isolates (NDV/Duck/A74/19, NDV/Duck/M147/19, NDV/Duck/BK43/19, and NDV/Duck/TD19/19) were classified as lentogenic NDVs, as both their ICPI values and F protein cleavage sites contained the amino acid motif characteristic for virulence in chickens [4]. Our results demonstrated that both NDV virulent or velogenic and avirulent or lentogenic strains were isolated from unvaccinated ducks. Moreover, these strains could be transmitted to chickens, ducks, and geese through naïve contact.

Molecular pathotyping was performed based on the amino acid sequences of the F0 protein proteolytic cleavage site motifs (residues 112-117) of the isolated NDV strains. This method is rapid and reliable for NDV pathotyping compared with MDT, intravenous pathogenicity index, or ICPI [20]. The F protein cleavage site is the main determinant of viral virulence; however, other proteins, such as HN, the polymerase complex P and L, and M, can have an impact on the virulence and virus replication in chickens [21-24]. Previously, the nucleotide sequences at the *F* gene cleavage site have been used to predict the pathotype of NDV [25]. In general, the nucleotide sequences at the cleavage site of NDV virulent strains have three basic amino acids (multi-basic cleavage site): Arginine (R) and lysine (K) at positions 112-116 and amino acid phenylalanine (F) at position 117. Conversely, avirulent strains have <3 basic amino acids (mono-basic cleavage site) at positions 112-116 and amino acid leucine (L) at position 117 [4,26]. In addition, the presence of the phenylalanine (F) residue at position 117 has been described as being a possible contributor to the neurological effects [27]. The results confirmed that in this study, two isolates shared the cleavage site motif RRQKRF (amino acids 112-117), which are characteristic for velogenic strains, and which share the same sequence of the *F* gene cleavage site with earlier NDV isolated from 2009 to 2012 in Indonesia [28], and other avirulent isolates sharing the cleavage site motif GRQGRL.

The amino acid sequence (Figure-4) and F protein mutations at the cleavage site were analyzed. The sample isolates exhibited arginine (R), and the dominant lentogenic strain exhibited glutamine (Q) at amino acid position 114; based on the study conducted by Samal *et al*. [29], the mutation from glutamine to arginine at amino acid 114 of NDV virulent strains demonstrated reduced pathogenicity and viral ­replication in 1-day-old chickens. The mutation of some amino acids that were not part of the cleavage site did not change the virulence of strains previously reported in swans and geese [30].

In Indonesia, most research has focused on NDVs isolated from chickens due to their importance as a food-producing species. However, there are several studies on NDV in ducks and pigeons. Ducks and pigeons exhibit variable susceptibility to different NDV strains [31-33]. Molecular epidemiology research demonstrated that most NDVs isolated from ducks and pigeons, in general, belong to genotypes II, III, VI, and IX [18,34].

The phylogenetic analysis revealed that these six isolates belonged to three different NDV genotypes. In Indonesia, ND outbreaks in vaccinated chicken flocks have been previously reported [13]. To date, genotype VII of NDV is predominant in the domestic poultry in Asia [35]. NDV isolates from duck or pigeon can infect chicken and may be transmitted to native chickens [36]. In addition, ducks and geese are usually asymptomatic, but some isolates (genotypes VII, VI, and I) have caused outbreaks among geese and domestic ducks from China [37-39]. The outbreaks that occurred in Sansui Sheldrake duck flocks in Guizhou Province, which caused about 30% mortality, were a result of a genotype VIId strain [40].

Clinical cases have also been described occasionally in ducks. Numerous NDV strains of differing virulence have been isolated from diseased and clinically healthy ducks [41]. Some isolates were pathogenic in ducks and geese, and in recent years, the number of natural ND cases in ducks has been gradually increasing [42]. This indicates that ducks may not be just reservoirs and carriers of NDV but also susceptible to some strains of the virus [43,44].

At present, vaccine strains LaSota, B1, Komarov, and V4 are widely used in Indonesia to control NDV infection. However, sporadic ND cases vaccinated with LaSota in commercial farms were previously reported in Indonesia [13]. However, it is possible that NDV strains responsible for sporadic ND outbreaks in vaccinated chickens can escape the immune responses and thus contribute to the emergence of new genotypes [45]. Therefore, the control of NDV in different avian species of poultry through vaccination still faces new challenges. To summarize, stricter biosecurity measures should be urgently applied in poultry management to reduce the transmission of NDV among species. In addition, it is important to enhance the regular monitoring of ducks, geese, and other waterfowl in the NDV geographic distribution. The present research provides important information on the epidemiology, diagnosis, and control of NDV in Indonesia, as well as the importance of supporting the investigation of transboundary animal diseases in developing countries.

## Conclusion

The results demonstrate that ducks from Indonesia carry various genotypes and pathotypes of NDV. The results also show that the NDV isolates from duck were previously identified in duck species but still do not cluster in the same phylogenetic group. Our findings indicate that NDV is still circulating in the environment. Such knowledge may be valuable for future studies to improve the control and diagnostic strategies of this disease.

## Authors’ Contributions

NP executed the work (collected samples, analyzed data, and prepared the manuscript). NP designed the study and drafted the manuscript under the guidance of FAR and RE. FAR, RE, SS, and JR revised the manuscript. All authors read and approved the final version of the manuscript.
